# Association of inflammation and nutrition-based indicators with chronic obstructive pulmonary disease and mortality

**DOI:** 10.1186/s41043-024-00709-x

**Published:** 2024-12-06

**Authors:** Kaiqiang Cao, Xiaoyang Miao, Xiaorong Chen

**Affiliations:** 1 Department of Emergency, the First Affiliated Hospital of Wenzhou Medical University, Ouhai District, Wenzhou, 325000 China; 2 Department of Intensive Care Unit, , the First Affiliated Hospital of Wenzhou Medical University, Wenzhou, 325000 China

**Keywords:** RAR, COPD, Mortality, NHANES

## Abstract

**Background:**

Inflammation and nutrition are strongly linked to respiratory diseases, but the link between inflammation and nutrition-based indicators and chronic obstructive pulmonary disease (COPD) and its mortality has not been reported.

**Methods:**

We recruited adults no younger than 20 years old from the NHANES 1999–2018. Inflammation and nutrition-based indicators included NAR, PNI, MAR, RAR, HALP, and ALI. COPD were assessed through a self-report questionnaire. Participants’ mortality rates were determined by association with the National Death Index.

**Results:**

A total of 46,572 individuals were collected in this study, including 1,549 COPD patients. NAR, MAR, and RAR were positively linked with the prevalence of COPD. However, PNI and HALP were negatively linked with the prevalence of COPD. In participants with COPD, the highest quartile of NAR (HR = 1.43 [1.04–1.97]), MAR (HR = 1.66 [1.23–2.26]), and RAR (HR = 2.45 [1.90–3.17]) were linked with an increased risk of all-cause mortality compared to the lowest quartile. However, the highest quartile of PNI (HR = 0.48 [0.38–0.61]) and HALP (HR = 0.56 [0.44–0.71]) were linked with a decreased risk of all-cause mortality compared to the lowest quartile. Randomized survival forests (RSF) showed that RAR had the strongest predictive power for all-cause mortality in COPD individuals among all indicators.

**Conclusion:**

We found that inflammation and nutrition-based indicators were linked to prognosis in COPD patients, with RAR having the highest predictive value.

**Supplementary Information:**

The online version contains supplementary material available at 10.1186/s41043-024-00709-x.

## Introduction

Chronic Obstructive Pulmonary Disease (COPD) is a common respiratory disease that poses a huge burden on global public health [1]. According to research data, COPD is the third most common fatal disease in the world, leading to approximately 3 million deaths annually [2]. Chronic Obstructive Pulmonary Disease (COPD) is a collective term for a group of lung diseases that share the common feature of pathological damage to lung tissue, leading to a decline in lung function [3]. COPD is mainly characterised by chronic cough, sputum, dyspnoea and chest tightness, as well as systemic symptoms such as weight reduction and loss of appetite [4]. COPD is mainly comprised of two types, chronic bronchitis and emphysema, and its pathogenesis is complex, often linked to environmental factors such as cigarette smoking, air pollution, and genes [5]. In addition to these known risk factors, inflammation and nutritional status may also be involved in the progression of COPD [6].

Inflammation is an essential feature of the pathophysiological process of COPD [7]. In the airways and lung tissues of COPD patients, chronic inflammatory responses are often present, including the release of cytokines, chemokines, and inflammatory mediators, which lead to narrowing of the airways and destruction of the alveolar [8]. These inflammatory responses not only aggravate the obstruction of the airways, but may also cause the occurrence of dyspnoea, thus aggravating the condition of COPD patients [9]. This inflammatory response is not limited to the lungs, but can also be detected in the peripheral blood as an increase in the number of pro-inflammatory cytokines, acute phase reactants, oxidative stress and activated inflammatory cells [10]. Nutritional status has been associated with the progression and prognosis of COPD [11]. COPD patients are often accompanied by abnormalities in nutritional status, including basal metabolic rate and metabolism [12]. Malnutrition not only affects immune function, but may also affect lung function, thus exacerbating COPD [13]. Therefore, it is significant to explore the link between inflammation and nutritional status and COPD to gain a new understanding of the pathogenesis of COPD.

In recent years, the relationship between inflammation and nutrition-related indicators and COPD has garnered significant attention from clinicians. While these composite indices are commonly used to assess the inflammatory and nutritional status of patients, most studies have focused on their inflammatory components, given the role of albumin as an acute phase protein. Few studies have systematically explored and compared the predictive value of these indices in relation to both COPD prevalence and all-cause mortality in COPD patients. The aim of this study was to evaluate whether these inflammation- and nutrition-based indicators are associated with COPD prevalence and to assess their utility in predicting all-cause mortality risk in COPD patients. Using data from adults who participated in the 1999–2018 National Health and Nutrition Examination Survey (NHANES), we analyzed the associations beween six composite inflammation- and nutrition-based indicators and COPD prevalence. Additionally, we investigated the value of these indicators in predicting all-cause mortality among COPD patients, recognizing the dual role of these markers in reflecting both inflammatory processes and nutritional status.

## Materials and methods

### Study population

NHANES is a large cross-sectional survey based on the U.S. population designed to collect data on the nutrition, diet, and health status of the nation’s population [14]. The study is conducted by the National Center for Health Statistics (NCHS) and has been collecting data biennially since 1971. NHANES uses a complex, stratified, multi-stage whole population sample to collect relevant data including demographic, socio-economic, and physiological assessments, laboratory tests, and other nutritional health information. The study was approved by the NCHS Ethics Review Board and all participants signed an informed consent form.

For the 1999–2018 NHANES, we excluded participants younger than 20 years old and without information on COPD related assessment data (*n* = 46,240), participants without data for inflammation and nutritional indicators (*n* = 7,133), and participants who were pregnant (*n* = 1,289). After excluding participants did not complete follow-up (*n* = 82), a total of 46,572 participants were used for analysis (Figure S1).

### Assessment of inflammation and nutrition-based indicators

Red cell distribution width (RDW) reflects the heterogeneity of erythrocyte volume and has traditionally been used in the differential diagnosis of anaemia [15]. Neutrophil-albumin ratio (NAR) is a composite indicator of peripheral blood neutrophil and lymphocyte counts, representing a state of systemic inflammation [16]. Prognostic nutritional index (PNI), a novel immunonutritional risk indicator calculated from albumin and total lymphocyte counts, was first proposed in 1984 and was initially used to assess cancer prognosis [17]. Monocyte-albumin ratio (MAR), calculated from the ratio of monocytes to albumin, reflects systemic inflammation and nutritional status [18]. Red cell distribution width-albumin ratio (RAR) is a newly identified risk marker and has been linked with prognosis in a wide range of inflammatory diseases [19]. Hemoglobin, albumin, lymphocyte, and platelet (HAlP) is a novel immunonutritional marker to predict clinical outcomes in oncology patients [20]. Advanced lung cancer inflammation index (ALI), originally used to assess systemic levels of inflammation in cancer patients, is a combination of body weight, albumin, and neutrophil-to-lymphocyte ratio index [21]. Table S1 demonstrates the calculation methods of combination in each inflammation and nutrition-based indicators.

### Assessment of COPD and all-cause mortality

Participants with COPD were defined as meeting at least one of the following criteria: (1) having ever been told by a healthcare provider that they had emphysema, or (2) being over 40 years old, having a history of smoking or a diagnosis of chronic bronchitis, and using medications commonly prescribed for COPD (such as selective phosphodiesterase-4 inhibitors, mast cell stabilizers, leukotriene modifiers, or inhaled corticosteroids) [22].

NHANES records deaths through linkage to the National Mortality Index, and follow-up ends on December 31, 2019. All-cause death was defined as the total death from all causes within a given period.

### Covariates

Information on age, sex, race, education, and marital status was collected. According to age, participants were divided into three groups (20–39 years, 40–59 years, and ≥ 60 years). According to marital status, they were divided into married/living with partner and single/divorced/widowed. The comprehensive definition of other covariates can be found in the supplementary materials, including family poverty income ratio (PIR, ≤ 1.0, 1.1–3.0, or > 3.0), smoking status (never, former, or current smoker), drinking status (nondrinker, former drinker, or current drinker), physical activity (inactive, insufficiently active, or active), healthy eating index (HEI), and Charlson comorbidity index (CCI) [23–28].

### Statistical analysis

The demographic descriptive statistics were calculated using sampling weights. Normally distributed continuous variables are described as means ± SEs, and continuous variables without a normal distribution are presented as medians [interquartile ranges]. Categorical variables are presented as numbers (percentages). Continuous variables were compared using Student’s t-test (for normal distribution) or Mann-Whitney U test (for non-normal distribution), while categorical variables were compared using the chi-square test. The correlation coefficients between inflammation and nutrition-based indicators and other parameters were calculated using Spearman’s correlation analysis. All analyses were performed using R (version 4.2.0) and *P*-values less than 0.05 were considered statistically significant.

Logistic regression analyses were used to examine the link between inflammation and nutrition-based indicators and the prevalence of COPD. COX proportional hazard regression analyses were used to assess the link of inflammation and nutrition-based indicators with all-cause mortality in COPD patients. Restricted cubic spline curve (RCS) is used to investigate whether there is a non-linear link in the above relationships. The log-rank test was performed to assess survival in various groups using Kaplan-Meier survival curves. The ability of these metrics to predict all-cause mortality in COPD patients was assessed by receiver operating characteristic curves (ROC). The usefulness of inflammation and nutrition-based indicators in predicting all-cause death in COPD patients was compared using a random subsistence forest method. In addition, we analysed the link between inflammation and nutrition-based indicators and other parameters with the prevalence of COPD and all-cause mortality.

## Results

### Baseline characteristics of the participants

Table 1 summarizes the initial traits of 46,572 individuals, including 1,549 COPD patients. The percentage of participants aged 20–39 years was 37%, 40–59 years was 38.13%, and ≥ 60 years was 24.87%. 51.98% were female and 48.92% were male. Participants with COPD were older, more likely to be non-Hispanic White, highly educated, medium economic level, less active, former or current smokers, and had highly CCI. Notably, NAR, MAR, RAR, and ALI were significantly higher in COPD patients, while PNI and HALP was significantly lower in COPD patients (*P* < 0.05). Table S1 presents baseline characteristics of participants with COPD according to all-cause mortality.

**Table 1:** Baseline characteristics of adult participants according to COPD in NHANES 1999–2018

Characteristics	Total	COPD		*P* value
		No (*n* = 45023)	Yes (*n* = 1549)	
Age, %				< 0.01
20–39 years	15,221(37.00)	15,185(38.06)	36(2.87)	
40–59 years	15,317(38.13)	14,845(38.11)	472(38.67)	
≥ 60 years	16,034(24.87)	14,993(23.83)	1041(58.46)	
Sex, %				0.04
Female	23,476(51.08)	22,736(50.97)	740(54.70)	
Male	23,096(48.92)	22,287(49.03)	809(45.30)	
Race/ethnicity, %				< 0.01
Non-Hispanic White	20,806(68.90)	19,792(68.50)	1014(81.95)	
Non-Hispanic Black	9413(10.55)	9164(10.66)	249(7.07)	
Other race	16,353(20.54)	16,067(20.84)	286(10.98)	
Marital status, %				< 0.01
Married/living with partner	18,446(35.94)	17,707(35.77)	739(41.18)	
Single/divorced/widowed	28,126(64.06)	27,316(64.23)	810(58.82)	
Education level, %				< 0.01
Below high school	12,537(17.17)	12,004(16.88)	533(26.35)	
High school	10,774(24.03)	10,388(23.95)	386(26.42)	
Above high school	23,261(58.80)	22,631(59.16)	630(47.23)	
Family PIR, %				< 0.01
≤ 1.0	9572(14.27)	9171(14.10)	401(19.79)	
1.1–3.0	19,712(36.20)	18,984(35.99)	728(43.04)	
> 3.0	17,288(49.53)	16,868(49.91)	420(37.16)	
Smoking status, %				< 0.01
Never smoker	25,126(53.61)	24,918(54.83)	208(14.34)	
Former smoker	11,608(24.78)	10,791(23.97)	817(51.01)	
Current smoker	9838(21.61)	9314(21.20)	524(34.65)	
Drinking status, %				0.12
Nondrinker	10,661(18.55)	10,306(18.47)	355(21.10)	
Low-to-moderate drinker	32,123(71.85)	31,069(71.94)	1054(69.01)	
Heavy drinker	3788(9.60)	3648(9.59)	140(9.90)	
Physical activity, %				< 0.01
Inactive	12,645(21.87)	11,975(21.30)	670(40.22)	
Insufficiently active	17,267(40.17)	16,797(40.46)	470(30.68)	
Active	16,660(37.96)	16,251(38.24)	409(29.10)	
HEI-2015 score	49.66(40.52,59.48)	49.70(40.57,59.48)	48.61(38.99,58.41)	0.04
CCI	0.85(0.01)	0.82(0.01)	2.08(0.07)	< 0.01
Inflammation and nutritional indicators				
Neutrophil, 10^3^/µL	4.00(3.20,5.10)	4.00(3.10,5.10)	4.60(3.50,5.70)	< 0.01
Lymphocyte, 10^3^/µL	2.00(1.60,2.50)	2.00(1.60,2.50)	2.00(1.50,2.50)	< 0.01
Monocyte, 10^3^/µL	0.50(0.40,0.70)	0.50(0.40,0.70)	0.60(0.50,0.70)	< 0.01
Hemoglobin, g/dL	14.40(13.40,15.40)	14.40(13.40,15.40)	14.20(13.20,15.20)	0.04
RDW, %	12.80(12.30,13.50)	12.80(12.30,13.50)	13.30(12.70,14.10)	< 0.01
Platelet, 10^3^/µL	246.00(210.00,290.00)	246.00(210.00,290.00)	252.00(209.00,303.00)	0.03
Serum albumin, g/L	43.00(41.00,45.00)	43.00(41.00,45.00)	42.00(40.00,44.00)	< 0.01
Body mass index, kg/m^2^	27.66(24.06,32.12)	27.62(24.04,32.07)	28.60(24.47,34.10)	< 0.01
Inflammation/nutrition-based indicators				
NAR	0.09(0.07,0.12)	0.09(0.07,0.12)	0.11(0.08,0.14)	< 0.01
PNI	53.50(50.50,56.50)	53.50(50.50,56.50)	51.50(48.50,55.00)	< 0.01
MAR	0.01(0.01,0.02)	0.01(0.01,0.02)	0.01(0.01,0.02)	< 0.01
RAR	0.30(0.28,0.33)	0.30(0.28,0.32)	0.32(0.30,0.36)	< 0.01
HALP score	50.58(38.95,65.67)	50.68(39.11,65.73)	47.04(33.38,62.84)	< 0.01
ALI	238.33(173.88,326.72)	237.33(173.48,324.76)	280.27(194.81,411.57)	< 0.01

Abbreviations: HEI-2015, Healthy Eating Index 2015; CCI, Charlson Comorbidity Index; RDW, red cell distribution width; NAR, neutrophil-albumin ratio; PNI, prognostic nutritional index; MAR, monocyte-albumin ratio; RAR, red cell distribution width-albumin ratio; HALP, hemoglobin, albumin, lymphocyte, and platelet; ALI, advanced lung cancer inflammation index. Normally distributed continuous variables are described as means ± SEs, and continuous variables without a normal distribution are presented as medians [interquartile ranges]. Categorical variables are presented as numbers (percentages). N reflect the study sample while percentages reflect the survey-weighted data

### The link between inflammation and nutrition-based indicators and COPD prevalence

In the crude model, we observed positive links between NAR, MAR, RAR, ALI, and the prevalence of COPD (Table 2). However, we observed negative links between PNI, HALP, and the prevalence of COPD. After adjusting for some confounding factors, including age, sex, race/ethnicity, marital status, education level, family PIR, drinking status, smoking status, physical activity, HEI, and CCI, these relationships remained statistically significant. Compared to the first quartile, the fourth quartile of NAR (OR = 1.71 [1.35–2.18]), MAR (OR = 1.38 [1.09–1.76]), and RAR (OR = 2.11 [1.65–2.70]) were positively linked with the prevalence of COPD after multivariable adjustment. However, the fourth quartile of PNI (OR = 0.75 [0.59–0.95]) and HALP (OR = 0.64 [0.53–0.77]) were negatively linked with the prevalence of COPD, compared to the first quartile. RCS analysis showed that the relationship between NAR, PNI, MAR and COPD prevalence was linear (P for non-linearity > 0.05), whereas the relationship between RAR, HALP and COPD prevalence was non-linear, with inflection points of 0.32 and 44.41 (*P* for non-linearity < 0.05) (Figure S2). We further analyzed the association between specific components of inflammation and nutritional status (e.g., neutrophils, monocytes, RDW, platelets, and serum albumin) and the prevalence of COPD (Table S3), finding a strong correlation with COPD prevalence.

**Table 2:** Logistic regression analysis of the relationship between inflammation/nutrition-based indicators and the prevalence of COPD among adults in NHANES 1999–2018

	Quartiles of inflammation/nutrition-based indicators				*P*_trend_
	OR	OR (95% CI)	OR (95% CI)	OR (95% CI)	
NAR					
Crude	1 [Reference]	1.31 (1.04–1.66)	1.79 (1.45–2.21)	2.93 (2.35–3.65)	< 0.01
Model 1	1 [Reference]	1.20 (0.95–1.52)	1.60 (1.29–1.98)	2.78 (2.23–3.47)	< 0.01
Model 2	1 [Reference]	1.06 (0.84–1.34)	1.23 (0.98–1.54)	1.71 (1.35–2.18)	< 0.01
PNI					
Crude	1 [Reference]	0.57 (0.47–0.69)	0.50 (0.42–0.60)	0.44 (0.36–0.54)	< 0.01
Model 1	1 [Reference]	0.72 (0.59–0.87)	0.77 (0.64–0.93)	0.85 (0.69–1.06)	0.06
Model 2	1 [Reference]	0.74 (0.60–0.92)	0.81 (0.66–0.99)	0.75 (0.59–0.95)	0.02
MAR					
Crude	1 [Reference]	1.09 (0.86–1.37)	1.64 (1.30–2.07)	2.41 (1.94-3.00)	< 0.01
Model 1	1 [Reference]	1.06 (0.84–1.35)	1.48 (1.17–1.86)	1.96 (1.57–2.45)	< 0.01
Model 2	1 [Reference]	0.98 (0.77–1.25)	1.26 (0.98–1.61)	1.38 (1.09–1.76)	< 0.01
RAR					
Crude	1 [Reference]	2.28 (1.82–2.85)	2.98 (2.36–3.77)	5.02 (4.01–6.29)	< 0.01
Model 1	1 [Reference]	1.63 (1.30–2.05)	1.89 (1.49–2.41)	3.04 (2.41–3.84)	< 0.01
Model 2	1 [Reference]	1.51 (1.19–1.90)	1.56 (1.23–1.97)	2.11 (1.65–2.70)	< 0.01
HALP					
Crude	1 [Reference]	0.61 (0.51–0.73)	0.57 (0.47–0.70)	0.58 (0.49–0.69)	< 0.01
Model 1	1 [Reference]	0.70 (0.59–0.84)	0.73 (0.59–0.89)	0.80 (0.66–0.95)	0.03
Model 2	1 [Reference]	0.71 (0.58–0.86)	0.69 (0.55–0.86)	0.64 (0.53–0.77)	< 0.01
ALI					
Crude	1 [Reference]	0.91 (0.71–1.17)	1.20 (0.97–1.48)	1.89 (1.54–2.32)	< 0.01
Model 1	1 [Reference]	0.82 (0.63–1.06)	1.01 (0.81–1.25)	1.43 (1.17–1.75)	< 0.01
Model 2	1 [Reference]	0.84 (0.64–1.09)	0.98 (0.79–1.22)	1.23 (0.99–1.53)	< 0.01

Abbreviations: NAR, neutrophil-albumin ratio; PNI, prognostic nutritional index; MAR, monocyte-albumin ratio; RAR, red cell distribution width-albumin ratio; HALP, hemoglobin, albumin, lymphocyte, and platelet; ALI, advanced lung cancer inflammation index; Model 1: Adjusted for age (20–39, 40–59, or ≥ 60 years), sex (male or female), and race/ethnicity (non-Hispanic White, non-Hispanic Black or other race); Model 2: Model 1 + marital status (married/living with partner, or single/divorced/widowed), education level (below high school, high school, or above high school), family PIR (≤ 1.0, 1.1–3.0, or > 3.0), drinking status (nondrinker, former drinker, or current drinker), smoking status (never smoker, former smoker, or current smoker, physical activity (inactive, insufficiently active, or active), HEI (in quartiles), and CCI (continous)

### Association of inflammation and nutrition-based indicators with all-cause mortality in participants with COPD

**Fig. 1:**
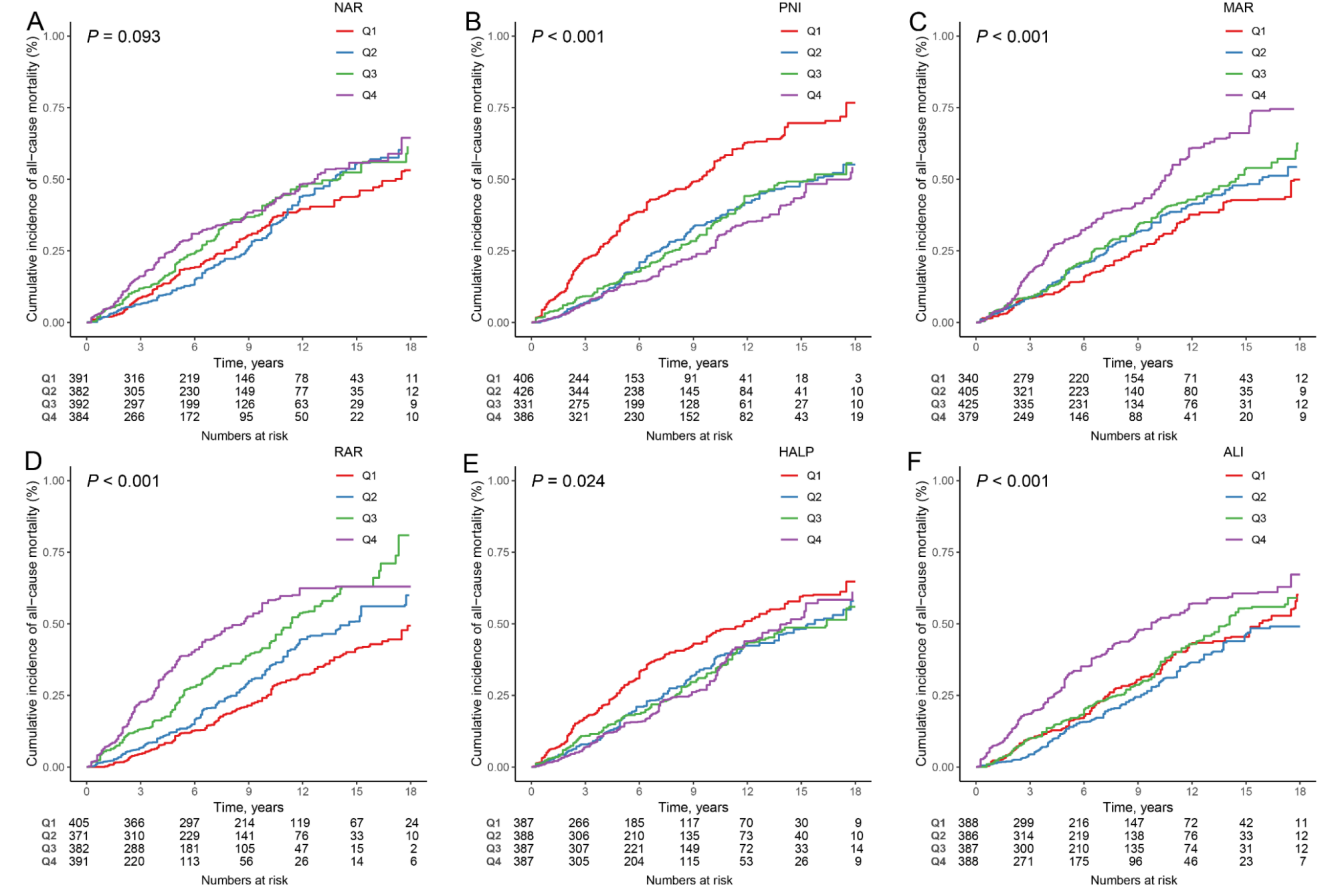
Kaplan-Meier survival curves for quartiles of inflammation and nutrition-based indicators (**A**: NAR; **B**: PNI; **C**: MAR; **D**: RAR; **E**: HALP; **F**: ALI) and all-cause mortality in patients with COPD. NAR, neutrophil-albumin ratio; PNI, prognostic nutritional index; MAR, monocyte-albumin ratio; RAR, red cell distribution width-albumin ratio; HALP, hemoglobin, albumin, lymphocyte, and platelet; ALI, advanced lung cancer inflammation index

**Fig. 2:**
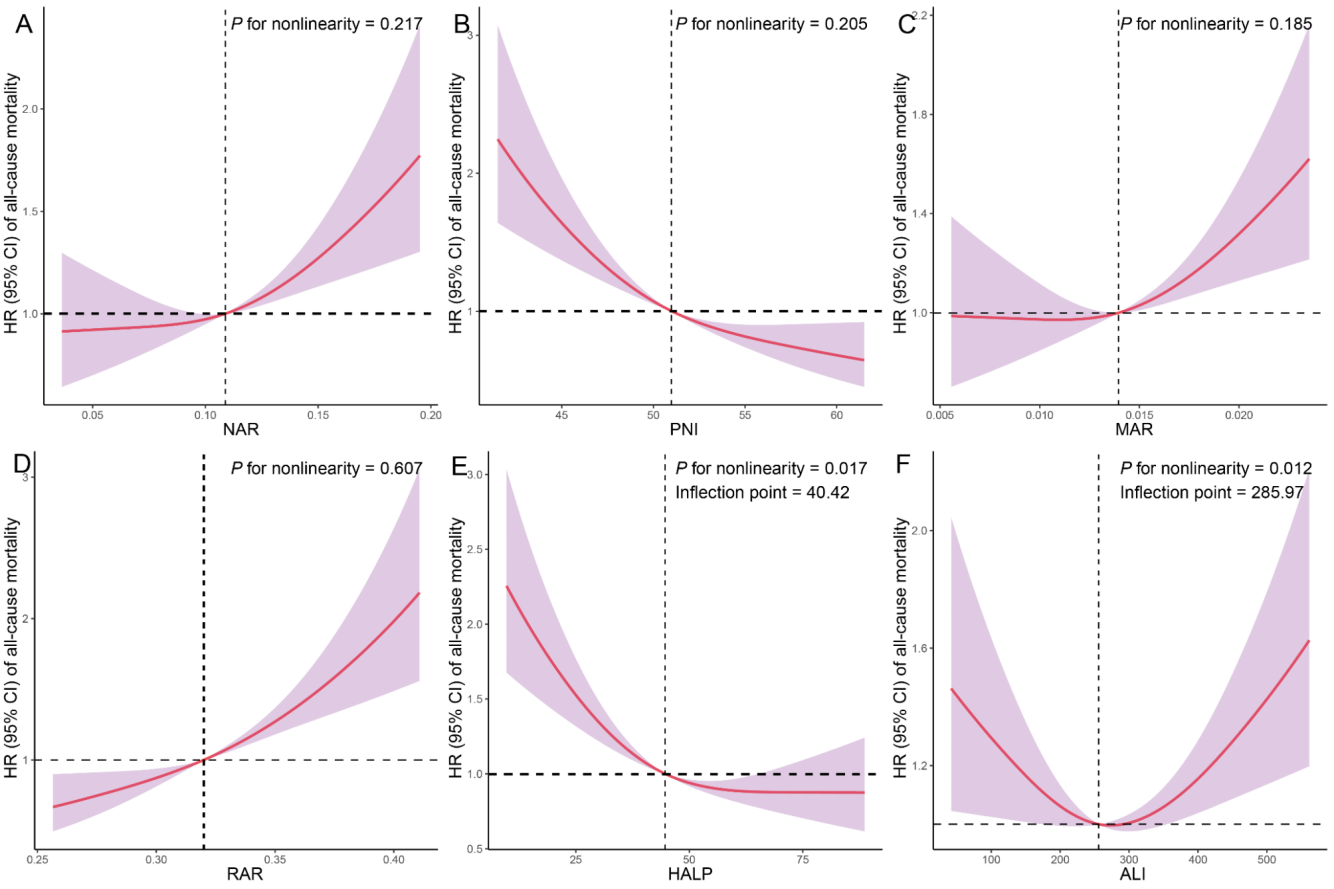
Restricted cubic spline (RCS) analysis with multivariate-adjusted associations of inflammation/nutrition-based indicators (**A**: NAR; **B**: PNI; **C**: MAR; **D**: RAR; **E**: HALP; **F**: ALI) with all-cause mortality in patients with COPD. Models are adjusted for age (20–39, 40–59, or ≥ 60 years), sex (male or female), race/ethnicity (non-Hispanic White, non-Hispanic Black or other race), marital status (married/living with partner, or single/divorced/widowed), education level (below high school, high school, or above high school), family PIR (≤ 1.0, 1.1–3.0, or > 3.0), drinking status (nondrinker, former drinker, or current drinker), smoking status (never smoker, former smoker, or current smoker), physical activity (inactive, insufficiently active, or active), HEI (in quartiles), and CCI (continous). NAR, neutrophil-albumin ratio; PNI, prognostic nutritional index; MAR, monocyte-albumin ratio; RAR, red cell distribution width-albumin ratio; HALP, hemoglobin, albumin, lymphocyte, and platelet; ALI, advanced lung cancer inflammation index; GNRI, geriatric nutrition risk index; CONUT, controlling nutritional status

During a median follow-up of 6.92 years, 657 all-cause deaths were observed. Kaplan-Meier survival curves for inflammation and nutrition-based indicators and all-cause mortality in COPD participants are shown in Fig. 1A-F. As can be seen, participants with COPD who had high NAR, MAR, and RAR had the highest risk of all-cause mortality compared with participants who did not have COPD (log-rank *P* < 0.001). However, participants with COPD who had high PNI and HALP had the lowest risk of all-cause mortality compared with participants who did not have COPD (log-rank *P* < 0.001). In the crude model and model 1, participants with COPD in the highest quartile of NAR, MAR, and RAR were associated with a significantly increased risk of all-cause mortality compared to those in the lowest quartile (Table 3). However, all-cause mortality was significantly decreased in COPD participants with high PNI and HALP compared with controls. In participants with COPD, the highest quartile of NAR (HR = 1.43 [1.04–1.97]), MAR (HR = 1.66 [1.23–2.26]), and RAR (HR = 2.45 [1.90–3.17]) were linked with an increased risk of all-cause mortality compared to the lowest quartile after multivariable adjustment. However, the highest quartile of PNI (HR = 0.48 [0.38–0.61]) and HALP (HR = 0.56 [0.44–0.71]) were linked with a decreased risk of all-cause mortality compared to the lowest quartile. RCS analyses showed that the relationship between NAR, PNI, MAR, RAR and all-cause mortality in COPD patients was linear (*P* for non-linearity > 0.05), whereas the relationship between HALP and all-cause mortality in COPD patients was non-linear, with inflection points of 0.32 and 40.42 (*P* for non-linearity < 0.05) **(**Fig. 2A-F**).** We also looked at the relationship between specific components of inflammation and nutritional status and all-cause mortality in COPD patients (Table **S4** and Figure **S3**).

**Table 3:** COX regression analysis of the relationship between inflammation/nutrition-based indicators and all-cause mortality among adults with COPD in NHANES 1999–2018

	Quartiles of inflammation/nutrition-based indicators				*P*_trend_
	HR	HR (95% CI)	HR (95% CI)	HR (95% CI)	
NAR					
Crude	1 [Reference]	1.06 (0.80–1.41)	1.27 (0.94–1.70)	1.46 (1.07–2.01)	0.01
Model 1	1 [Reference]	0.98 (0.72–1.33)	1.34 (0.97–1.85)	1.65 (1.20–2.27)	< 0.01
Model 2	1 [Reference]	0.87 (0.64–1.17)	1.15 (0.81–1.63)	1.43 (1.04–1.97)	0.01
PNI					
Crude	1 [Reference]	0.51 (0.39–0.66)	0.50 (0.37–0.66)	0.41 (0.32–0.53)	< 0.01
Model 1	1 [Reference]	0.56 (0.44–0.71)	0.52 (0.40–0.69)	0.52 (0.41–0.67)	< 0.01
Model 2	1 [Reference]	0.56 (0.45–0.71)	0.50 (0.37–0.67)	0.48 (0.38–0.61)	< 0.01
MAR					
Crude	1 [Reference]	1.23 (0.87–1.73)	1.33 (0.99–1.79)	2.08 (1.54–2.80)	< 0.01
Model 1	1 [Reference]	1.03 (0.73–1.46)	1.14 (0.86–1.51)	1.70 (1.28–2.25)	< 0.01
Model 2	1 [Reference]	1.08 (0.76–1.55)	1.08 (0.79–1.48)	1.66 (1.23–2.26)	< 0.01
RAR					
Crude	1 [Reference]	1.39 (1.06–1.82)	2.03 (1.51–2.72)	2.86 (2.13–3.86)	< 0.01
Model 1	1 [Reference]	1.35 (1.05–1.75)	1.66 (1.23–2.23)	2.77 (2.10–3.67)	< 0.01
Model 2	1 [Reference]	1.34 (1.04–1.74)	1.50 (1.12–2.01)	2.45 (1.90–3.17)	< 0.01
HALP					
Crude	1 [Reference]	0.71 (0.55–0.93)	0.68 (0.48–0.97)	0.65 (0.47–0.91)	0.02
Model 1	1 [Reference]	0.68 (0.53–0.86)	0.64 (0.48–0.87)	0.68 (0.53–0.86)	< 0.01
Model 2	1 [Reference]	0.63 (0.50–0.79)	0.61 (0.45–0.81)	0.56 (0.44–0.71)	< 0.01
ALI					
Crude	1 [Reference]	0.81 (0.60–1.10)	1.05 (0.81–1.38)	1.69 (1.25–2.28)	< 0.01
Model 1	1 [Reference]	0.85 (0.64–1.13)	0.87 (0.67–1.14)	1.43 (1.05–1.96)	0.01
Model 2	1 [Reference]	0.82 (0.64–1.06)	0.89 (0.69–1.14)	1.36 (0.99–1.85)	0.01

Abbreviations: NAR, neutrophil-albumin ratio; PNI, prognostic nutritional index; MAR, monocyte-albumin ratio; RAR, red cell distribution width-albumin ratio; HALP, hemoglobin, albumin, lymphocyte, and platelet; ALI, advanced lung cancer inflammation index; Model 1: Adjusted for age (20–39, 40–59, or ≥ 60 years), sex (male or female), and race/ethnicity (non-Hispanic White, non-Hispanic Black or other race); Model 2: Model 1 + marital status (married/living with partner, or single/divorced/widowed), education level (below high school, high school, or above high school), family PIR (≤ 1.0, 1.1–3.0, or > 3.0), drinking status (nondrinker, former drinker, or current drinker), smoking status (never smoker, former smoker, or current smoker), physical activity (inactive, insufficiently active, or active), HEI (in quartiles), and CCI (continous)

### The value of inflammation and nutrition-based indicators in assessing the risk of all-cause mortality in patients with COPD

**Fig. 3:**
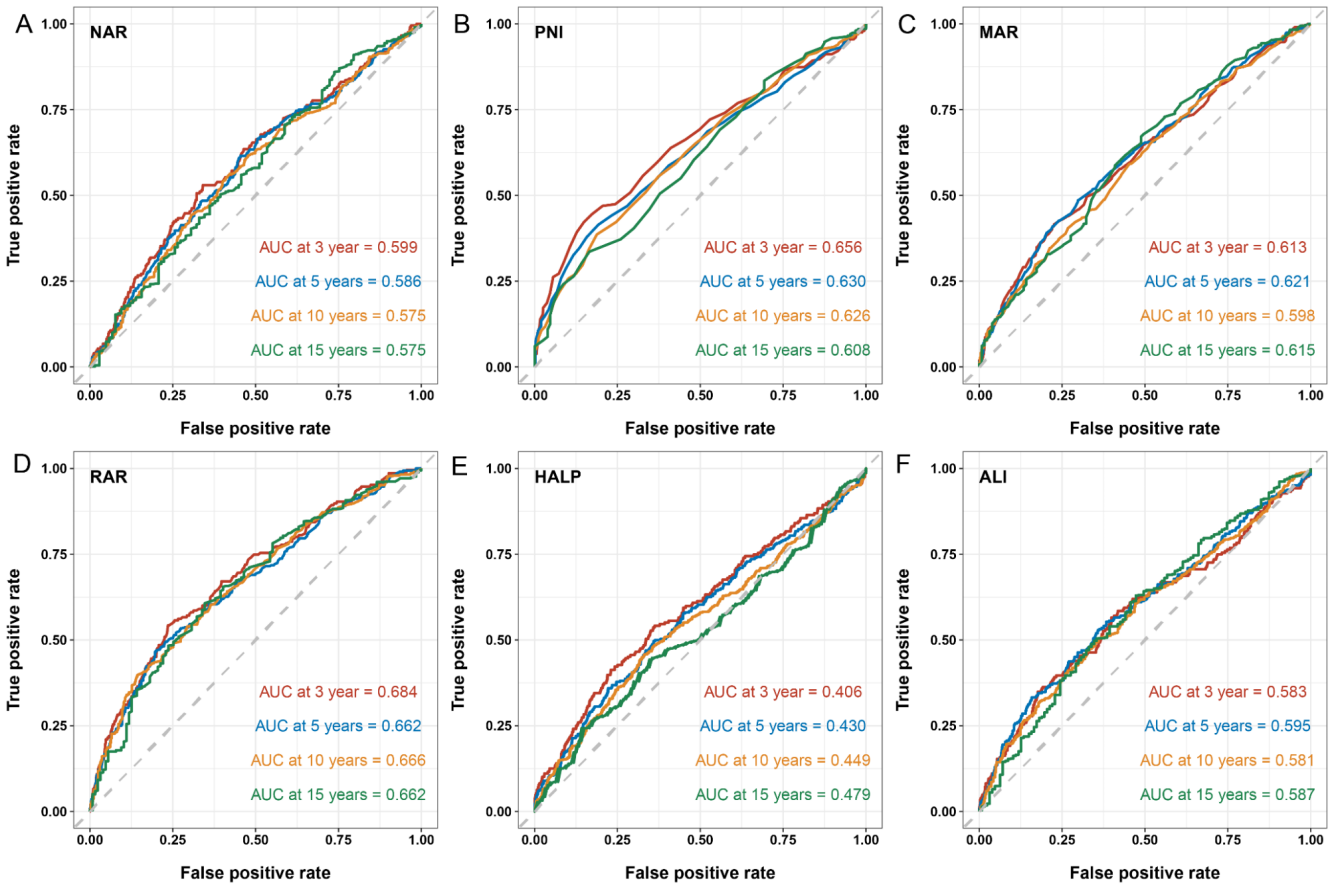
Predictive value of time-dependent ROC assessment of inflammation and nutrition-based indicators (**A**: NAR; **B**: PNI; **C**: MAR; **D**: RAR; **E**: HALP; **F**: ALI) for 3-, 5-, and 10-year all-cause mortality. NAR, neutrophil-albumin ratio; PNI, prognostic nutritional index; MAR, monocyte-albumin ratio; RAR, red cell distribution width-albumin ratio; HALP, hemoglobin, albumin, lymphocyte, and platelet; ALI, advanced lung cancer inflammation index

**Fig. 4:**
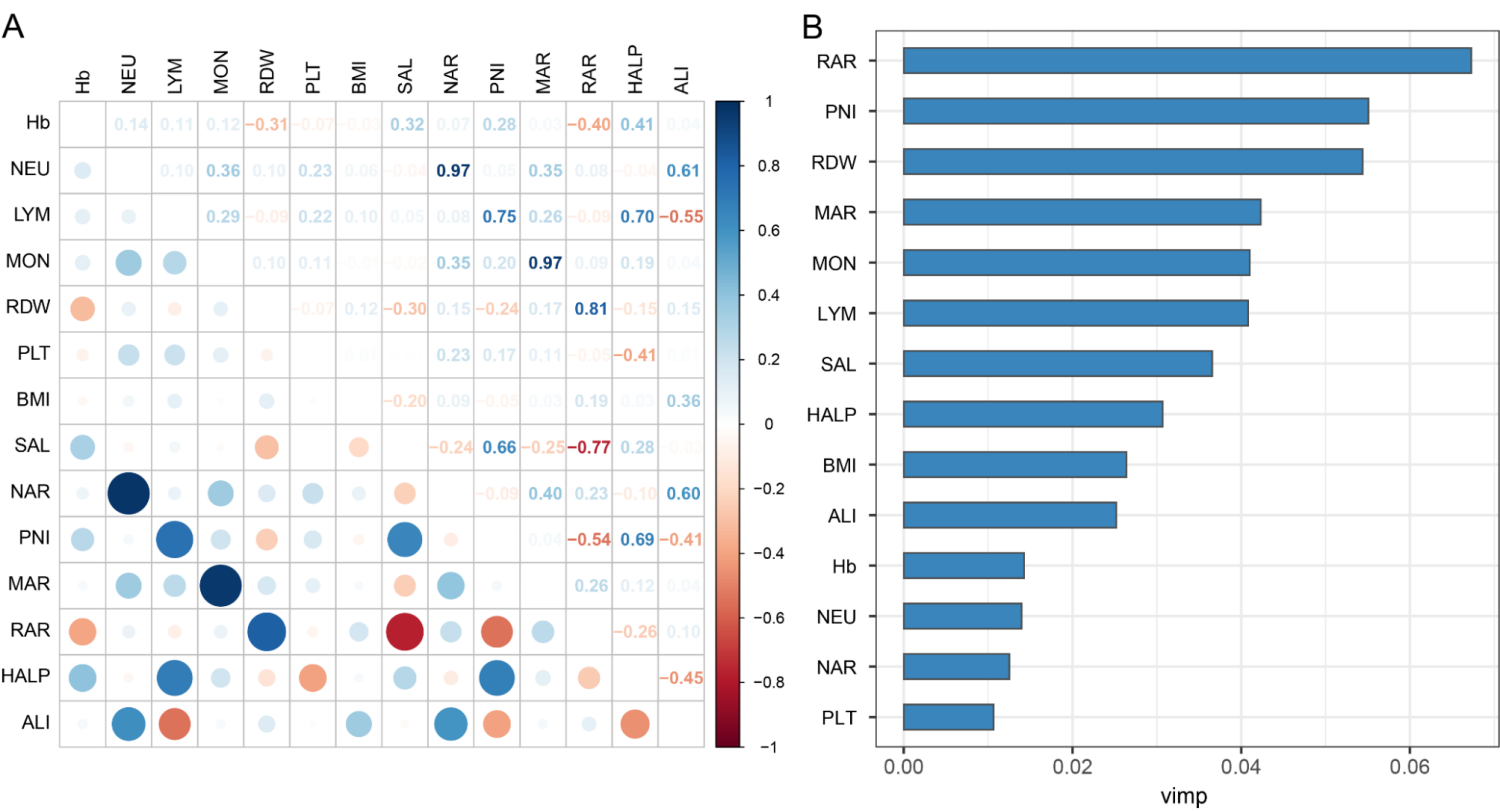
Prognostic value of inflammation and nutrition-based indicators. (**A**) Spearman correlation analysis was used to calculate the correlation coefficients among inflammation and nutrition-based indicators and other parameters. (**B**) A random subsistence forest method was used to compare the value of inflammation and nutrition-based indicators and other parameters in predicting all-cause mortality in COPD patients

Based on the ROC curve analysis, we found that RAR was the strongest predictor of all-cause mortality in COPD patients (AUC at 3 years = 0.68) (Fig. 3A-F). The correlations between inflammation and nutrition-based indicators and other parameters were showed in Fig. 4A. We found a strong positive link between PNI and HALP (*r* = 0.69) and a strong negative link between PNI and RAR (*r*=-0.54). Among all inflammation and nutrition-based indicators and other parameters, RAR has the highest value in predicting all-cause mortality in COPD patients (Fig. 4B).

## Discussion

A total of 46,572 individuals were collected in this study, including 1,549 COPD patients. We found that NAR, MAR, and RAR were positively linked with the prevalence of COPD. However, PNI and HALP were negatively linked with the prevalence of COPD. In participants with COPD, high NAR, MAR, and RAR were linked with an increased risk of all-cause mortality. However, high PNI and HALP were linked with a decreased risk of all-cause mortality. Among these, RAR is the strongest inflammation and nutrition-based indicator to predict the risk of death in COPD patients.

Poor nutritional status and systemic inflammatory response are known to be linked to poor prognosis in COPD patients [29]. Of the six inflammation and nutrition-based indicators we examined, several have been shown to be linked to COPD. PNI is an indicator of immunonutritional status. Suzuki’s study showed that a low PNI was linked to worsening of the disease in COPD patients [30]. HALP is a new indicator of systemic inflammation and nutritional status. Han et al. showed that low HALP scores were linked to an increased risk of death in patients with acute exacerbations of chronic obstructive pulmonary disease (AECOPD) [31]. In our study, compared to the first quartile, COPD prevalence increased by 171.4%, 138.2%, and 210.7% in the fourth quartile of NAR, MAR, and RAR, respectively. However, COPD prevalence decreased by 24.9% and 26.2% in the fourth quartile of PNI and HALP, respectively. In participants with COPD, compared to the lowest quartile, all-cause mortality increased by 143.3%, 166.4%, and 245.3% in the highest quartile of NAR, MAR, and RAR, respectively. However, all-cause mortality decreased by 51.9% and 44.3% in the fourth quartile of PNI and HALP, respectively. It is noteworthy that RAR not only shows a high correlation with the prevalence of COPD, but is also the best predictor of the risk of death in COPD patients.

RAR is defined as the ratio of RDW to albumin [32]. Previous studies have shown that high RDW and hypoalbuminaemia are markers of poor prognosis in patients with COPD. RDW reflects the degree of heterogeneity in erythrocyte volume and has been found to link to the prognosis of a variety of respiratory diseases [15]. Early studies have shown that elevated RDW is linked to the severity of COPD and the risk of mortality [33, 34]. Epstein et al. also confirmed that RDW is an independent prognostic factor for poor outcome in AECOPD patients [35]. Hypoxia is a common complication in patients with advanced COPD. Acute episodes of hypoxia can stimulate the production of erythropoietin, which induces erythrocytosis and may contribute to an increase in RDW in COPD patients [36]. Serum albumin levels are linked to the nutritional status of the organism and play an essential role in the maintenance of body homeostasis [37]. COPD is a chronic inflammatory disease and albumin is susceptible to oxidation under conditions of oxidative stress and chronic inflammation, leading to marked hypoalbuminaemia [38]. Serum albumin levels are a valid parameter for assessing post-hospital mortality and long-term survival in patients with AECOPD [39]. Lower albumin was identified as one of the strongest predictors of mortality in patients with AECOPD in a retrospective cohort study that included 574 participants [40].

RAR has been linked to clinical results in a range of respiratory illnesses. Yoo et al. showed that RAR was significantly linked to 60-day mortality in patients with acute respiratory distress syndrome [41]. Jeong et al. demonstrated that in critically ill patients with pneumonia, a higher RAR ratio was linked to a significantly increased 28-day mortality rate [42]. Currently, the link between RAR and COPD is controversial. Eraslan et al. showed that RAR was higher in COPD patients who were hospitalised for 10 days or more, but there was no significant correlation with 30-day mortality [43]. However, their study sample size was small and susceptible to selective bias. Qiu et al. found that higher RAR was linked to an increased risk of death in COPD patients in the intensive care unit. They demonstrated that RAR can independently predict in-hospital mortality in critically ill COPD patients [44]. Our study also found that RAR was more effective than other inflammation and nutrition-based indicators in predicting mortality in COPD patients. We hypothesised that the mechanism by which RAR predicts mortality in COPD may be related to hypoxia, inflammation and malnutrition. COPD patients suffer from long-term chronic inflammation, and the systemic inflammatory process can lead to catabolic stress in the patients [45]. Under the influence of systemic inflammatory stress, COPD patients are at significantly increased risk of malnutrition [46]. Malnutrition in COPD patients leads to impairment of respiratory muscle function, progression of disease severity, and also has a negative impact on mortality rates [47].

This study has several strengths. First, this study used a representative sample of the US population, and all measurements were performed according to a standardised protocol, which is highly persuasive. Second, we not only examined the links between the six inflammation and nutrition-based indicators and the prevalence of COPD, but also conducted prognostic analyses to explore the links between the inflammation and nutrition-based indicators and all-cause mortality in patients with COPD. Although this study found an association between inflammation and nutrition and COPD and its all-cause mortality, we have to acknowledge some limitations of the study. First, the data for this study were extracted from a public database and were limited to the US population. Second, this study only analysed single-measurement inflammation and nutrition-based indicators and did not analyse their dynamically evolving values, ignoring the impact of dynamic changes in these indicators. Third, the absence of consistent data on C-reactive protein (CRP) or other acute phase reactants across all NHANES survey cycles limited our ability to directly account for acute inflammation in the analysis. Therefore, the potential effects of unmeasured acute inflammatory responses cannot be entirely ruled out. Fourth, the specific causes of COPD in participants, particularly those diagnosed before age 39, could not be confirmed. Factors such as alpha-1 antitrypsin deficiency may have contributed to early-onset COPD, potentially influencing the interpretation of the findings. Finally, differentiating between asthma and COPD in this population-based study posed a challenge due to the overlapping clinical features of these conditions. Although we employed a multi-criteria approach based on age, smoking history, self-reported emphysema, chronic bronchitis, and medication use, the possibility of misclassification remains, potentially introducing inclusion bias. Future studies using more precise diagnostic criteria or longitudinal data could help mitigate this issue and improve differentiation between these respiratory conditions.

## Conclusion

This study demonstrated that inflammation and nutrition-based indicators are significantly associated with the prevalence of COPD. High NAR, MAR, RAR, and ALI were positively linked with the prevalence of COPD, whereas high PNI and HALP showed an inverse association. Among individuals with COPD, higher levels of NAR, MAR, and RAR were associated with an increased risk of all-cause mortality, while higher levels of PNI and HALP were linked to a decreased risk. Notably, RAR was identified as the strongest predictor of mortality risk among all indicators.

## Electronic supplementary material

Below is the link to the electronic supplementary material.

Supplementary Material 1

## Data Availability

NHANES data described in this manuscript are available at https://wwwn.cdc.gov/nchs/nhanes/..
