# Supplementary material for: Association of inflammation and nutrition-based indicators with chronic obstructive pulmonary disease and mortality
Source: J Health Popul Nutr. 2024 Dec 6;43:209. doi: 10.1186/s41043-024-00709-x (PMC11622568; doi:10.1186/s41043-024-00709-x)
Supplement: Supplementary file 1 — Supplementary Material 1 [file 41043_2024_709_MOESM1_ESM.docx]

**Online Supplementary Material**

**Association of Inflammation and Nutrition-Based Indicators with Chronic Obstructive Pulmonary Disease and Mortality**

**Figure S1.** Flowchart of the study

**Table S1.** Calculation methods of combination in each nutrition/inflammation-based indicator.

**Table S2.** Baseline characteristics of adult participants with COPD according to all-cause mortality in NHANES 1999–2018.

**Table S3.** Logistic regression analysis of the relationship between inflammation and nutritional indicators and the prevalence of COPD among adults in NHANES 1999–2018.

**Figure S2.** Restricted cubic spline (RCS) analysis with multivariate-adjusted associations of inflammation/nutrition‐based indicator with the prevalence of COPD among adults in NHANES 1999–2018.

**Table S4.** COX regression analysis of the relationship between inflammation and nutritional indicators and all-cause mortality among adults with COPD in NHANES 1999–2018.

**Figure S3.** Kaplan-Meier survival curves for quartiles of inflammation and nutrition indicators and mortality in adults.

**Materials and methods: Other Covariates**

This section provides a comprehensive definition of potential confounding variables including family poverty income ratio (PIR, ≤1.0, 1.1–3.0, or >3.0), smoking status (never, former, or current smoker), drinking status (nondrinker, former drinker, or current drinker), physical activity (inactive, insufficiently active, or active), healthy eating index (HEI), and Charlson comorbidity index (CCI).

***Family poverty income ratio*** Income was assessed using the poverty income ratio (PIR, the ratio of family income divided by a poverty threshold specific for family size using guidelines from the US Department of Health and Human Services) and categorized as ≤1.0, 1.1-3.0 and >3.0 [1].

***Smoking status*** Never smokers were classified as those who reported smoking <100 cigarettes during their lifetime. Those who smoked >100 cigarettes in their lifetime were considered as current smokers, and those who smoked >100 cigarettes and had quit smoking were considered as former smokers [2].

***Drinking status*** Drinking status was classified as nondrinker, low-to-moderate drinker (<2 drinks/day in men and <1 drink/day in women), or heavy drinker (≥2 drinks/day in men and ≥1 drinks/day in women) [2].

***Physical activity*** Physical activity was categorized as inactive group (no leisure-time physical activity), insufficiently active group (leisure time moderate activity 1–5 times per week with MET ranging from 3 to 6 or leisure-time vigorous activity 1–3 times per week with MET >6), or active group (those who had more leisure-time moderate-or-vigorous activity than above) [3].

***Healthy Eating Index*** The Healthy Eating Index (HEI) is a measure calculated from 24-hour dietary recall data to assess diet quality based on the 2015–2020 Dietary Guidelines for Americans (DGA) [4]. It comprises 13 subgroups, with a total possible score of 100. Nine components evaluate adequacy (higher intakes contribute to a higher score) including total fruits, whole fruits, total vegetables, greens and beans, whole grains, dairy, total protein foods, seafood and plant proteins, and fatty acids. The remaining four components assess moderation (lower intakes yield a higher score), covering refined grains, sodium, added sugars, and saturated fats. The HEI offers a comprehensive framework for understanding dietary patterns and their relationship to health outcomes, with scores reflecting adherence to key dietary recommendations.

***Charlson Comorbidity Index*** The Charlson Comorbidity Index (CCI) is a method used to quantify an individual's overall health status by summing the scores assigned to various diseases [5]. It assumes respondents without reported diseases as healthy, assigning a zero value to unreported conditions. This scoring approach follows the methodology established by Zhao et al. in prior research [6], ensuring consistency in the assessment of comorbidities.

**References**

1. **Services USDoHaH. Poverty Guidelines, Research, and Measurement** [<http://aspe.hhs.gov/POVERTY/index.shtml>.]

2. Qiu Z, Chen X, Geng T, Wan Z, Lu Q, Li L, Zhu K, Zhang X, Liu Y, Lin X *et al*: **Associations of Serum Carotenoids With Risk of Cardiovascular Mortality Among Individuals With Type 2 Diabetes: Results From NHANES**. *Diabetes Care* 2022, **45**(6):1453-1461.

3. Beddhu S, Baird BC, Zitterkoph J, Neilson J, Greene T: **Physical activity and mortality in chronic kidney disease (NHANES III)**. *Clin J Am Soc Nephrol* 2009, **4**(12):1901-1906.

4. Kirkpatrick SI, Reedy J, Krebs-Smith SM, Pannucci TE, Subar AF, Wilson MM, Lerman JL, Tooze JA: **Applications of the Healthy Eating Index for Surveillance, Epidemiology, and Intervention Research: Considerations and Caveats**. *J Acad Nutr Diet* 2018, **118**(9):1603-1621.

5. Kim CY, Sivasundaram L, LaBelle MW, Trivedi NN, Liu RW, Gillespie RJ: **Predicting adverse events, length of stay, and discharge disposition following shoulder arthroplasty: a comparison of the Elixhauser Comorbidity Measure and Charlson Comorbidity Index**. *J Shoulder Elbow Surg* 2018, **27**(10):1748-1755.

6. Zhao H, Pan Y, Wang C, Guo Y, Yao N, Wang H, Li B: **The Effects of Metal Exposures on Charlson Comorbidity Index Using Zero-Inflated Negative Binomial Regression Model: NHANES 2011-2016**. *Biol Trace Elem Res* 2021, **199**(6):2104-2111.

**
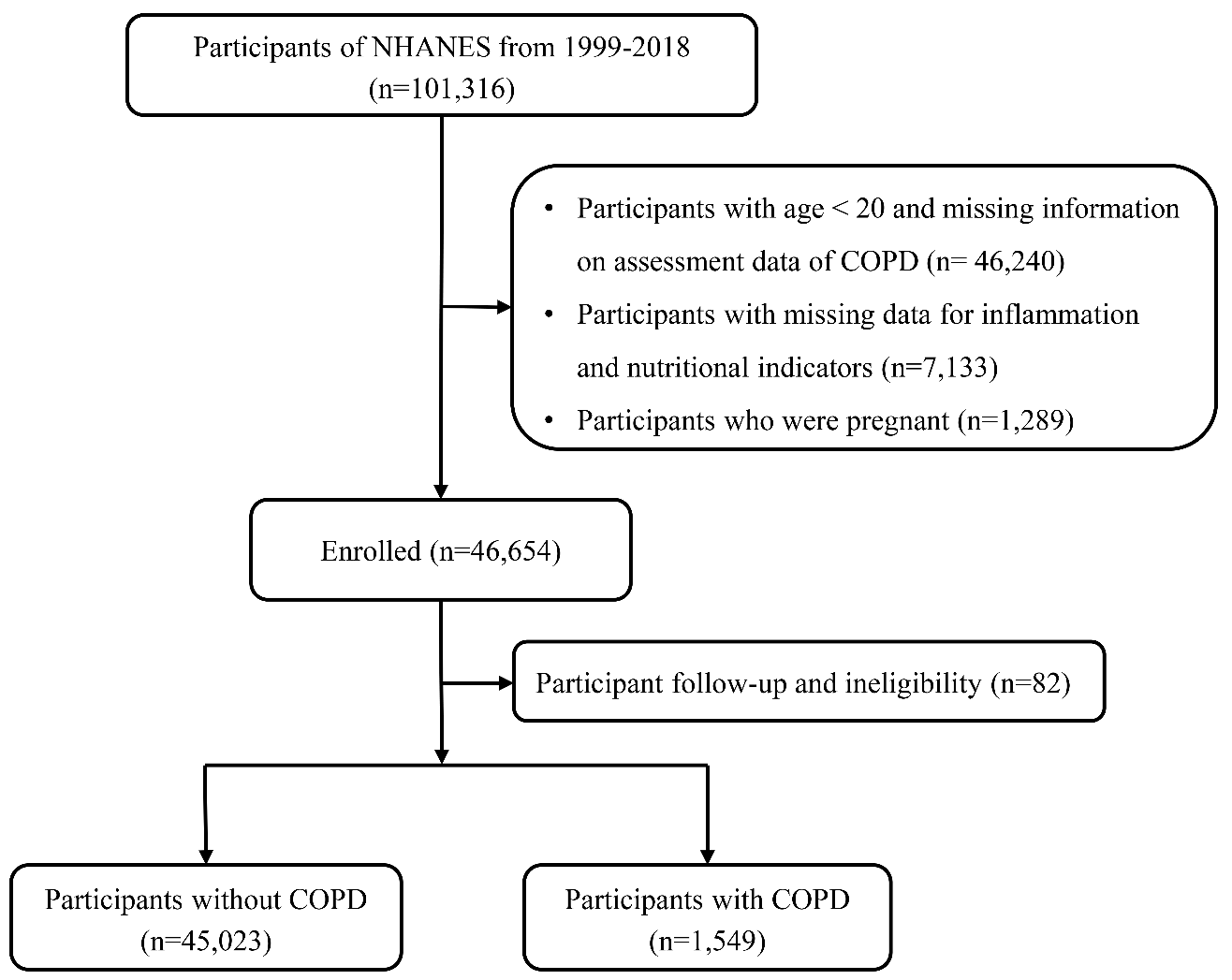
**

**Figure S1.** Flowchart of the study

**Table S1.** Calculation methods of combination in each nutrition/inflammation-based indicator.

| Indicators | Definition or calculation formula |
| --- | --- |
| NAR | neutrophil count (×10^9^)/ albumin (g/ L) |
| PNI | albumin (g/L) + 5×lymphocyte count (×10^9^) |
| MAR | monocyte count (×10^9^)/ albumin (g/ L) |
| RAR | RDW (%)/ albumin (g/ L) |
| HALP score | [hemoglobin (g/L) × albumin (g/L) × lymphocytes (×10^9^)]/platelets (×10^9^) |
| ALI | BMI (kg/m^2^) × albumin(g/dl)/NLR (×10^9^) |

**Table S2.** Baseline characteristics of adult participants with COPD according to all-cause mortality in NHANES 1999–2018.

| Characteristics | Total | All-cause Mortality | | P value |
| --- | --- | --- | --- | --- |
|  |  | No (n=892) | Yes (n=657) |  |
| Age, % |  |  |  | <0.01 |
| 20-39 years | 36(2.87) | 33(3.96) | 3(0.93) |  |
| 40-59 years | 472(38.67) | 381(49.89) | 91(18.59) |  |
| ≥60 years | 1041(58.46) | 478(46.16) | 563(80.48) |  |
| Sex, % |  |  |  | <0.01 |
| Female | 740(54.70) | 484(58.64) | 256(47.63) |  |
| Male | 809(45.30) | 408(41.36) | 401(52.37) |  |
| Race/ethnicity, % |  |  |  | <0.01 |
| Non-Hispanic White | 1014(81.95) | 519(78.61) | 495(87.91) |  |
| Non-Hispanic Black | 249(7.07) | 166(7.89) | 83(5.61) |  |
| Other race | 286(10.98) | 207(13.50) | 79(6.48) |  |
| Marital status, % |  |  |  | 0.01 |
| Married/living with partner | 739(41.18) | 413(37.94) | 326(46.98) |  |
| Single/divorced/widowed | 810(58.82) | 479(62.06) | 331(53.02) |  |
| Education level, % |  |  |  | <0.01 |
| Below high school | 533(26.35) | 272(22.54) | 261(33.17) |  |
| High school | 386(26.42) | 225(27.11) | 161(25.17) |  |
| Above high school | 630(47.23) | 395(50.35) | 235(41.66) |  |
| Family PIR, % |  |  |  | <0.01 |
| ≤1.0 | 401(19.79) | 239(18.50) | 162(22.11) |  |
| 1.1–3.0 | 728(43.04) | 384(39.01) | 344(50.26) |  |
| >3.0 | 420(37.16) | 269(42.49) | 151(27.63) |  |
| Smoking status, % |  |  |  | <0.01 |
| Never smoker | 208(14.34) | 143(16.86) | 65(9.83) |  |
| Former smoker | 817(51.01) | 429(47.93) | 388(56.51) |  |
| Current smoker | 524(34.65) | 320(35.20) | 204(33.65) |  |
| Drinking status, % |  |  |  | 0.70 |
| Nondrinker | 355(21.10) | 205(20.29) | 150(22.54) |  |
| Low-to-moderate drinker | 1054(69.01) | 603(69.51) | 451(68.11) |  |
| Heavy drinker | 140(9.90) | 84(10.20) | 56(9.35) |  |
| Physical activity, % |  |  |  | <0.01 |
| Inactive | 670(40.22) | 326(33.87) | 344(51.58) |  |
| Insufficiently active | 470(30.68) | 296(33.25) | 174(26.09) |  |
| Active | 409(29.10) | 270(32.88) | 139(22.33) |  |
| HEI-2015 score | 48.61(38.99,58.41) | 48.10(38.83,58.67) | 49.28(39.66,58.40) | 0.39 |
| CCI | 2.08(0.07) | 1.91(0.09) | 2.39(0.10) | <0.01 |
| Inflammation and nutritional indicators | |  |  |  |
| Neutrophil, 103/μL | 4.60(3.50,5.70) | 4.50(3.50,5.70) | 4.70(3.70,5.80) | 0.10 |
| Lymphocyte, 103/μL | 2.00(1.50,2.50) | 2.10(1.70,2.60) | 1.80(1.30,2.40) | <0.01 |
| Monocyte, 103/μL | 0.60(0.50,0.70) | 0.60(0.50,0.70) | 0.60(0.50,0.80) | 0.04 |
| Hemoglobin, g/dL | 14.20(13.20,15.20) | 14.20(13.20,15.20) | 14.20(13.20,15.20) | 0.64 |
| RDW, % | 13.30(12.70,14.10) | 13.30(12.60,14.10) | 13.40(12.70,14.30) | 0.10 |
| Platelet, 103/μL | 252.00(209.00,303.00) | 252.00(215.00,302.00) | 252.00(198.00,304.00) | 0.30 |
| Serum albumin, g/L | 42.00(40.00,44.00) | 42.00(40.00,44.00) | 42.00(39.00,44.00) | 0.69 |
| Body mass index, kg/m2 | 28.60(24.47,34.10) | 29.20(25.13,34.40) | 27.62(23.49,33.08) | <0.01 |
| Inflammation/nutrition‐based indicators | |  |  |  |
| NAR | 0.11(0.08,0.14) | 0.11(0.08,0.14) | 0.12(0.09,0.14) | 0.07 |
| PNI | 51.50(48.50,55.00) | 52.50(49.00,55.50) | 51.00(47.50,54.00) | <0.01 |
| MAR | 0.01(0.01,0.02) | 0.01(0.01,0.02) | 0.01(0.01,0.02) | 0.04 |
| RAR | 0.32(0.30,0.36) | 0.32(0.30,0.35) | 0.32(0.30,0.36) | 0.07 |
| HALP score | 47.04(33.38,62.84) | 48.68(36.16,64.87) | 42.37(30.22,59.11) | <0.01 |
| ALI | 280.27(194.81,411.57) | 268.49(189.61,381.10) | 310.08(196.80,460.00) | <0.01 |
| Follow-up time, years | 6.92(3.58,11.17) | 7.83(4.25,12.50) | 5.17(2.58, 9.33) | <0.01 |

Abbreviations: HEI-2015, Healthy Eating Index 2015; CCI, Charlson Comorbidity Index; RDW, red cell distribution width; NAR, neutrophil-albumin ratio; PNI, prognostic nutritional index; MAR, monocyte-albumin ratio; RAR, red cell distribution width-albumin ratio; HALP, hemoglobin, albumin, lymphocyte, and platelet; ALI, advanced lung cancer inflammation index.

Normally distributed continuous variables are described as means ± SEs, and continuous variables without a normal distribution are presented as medians [interquartile ranges]. Categorical variables are presented as numbers (percentages). N reflect the study sample while percentages reflect the survey-weighted data.

**Table S3.** Logistic regression analysis of the relationship between inflammation and nutritional indicators and the prevalence of COPD among adults in NHANES 1999–2018.

|  | Quartiles of inflammation and nutrition indicators | | | | *P_trend_* |
| --- | --- | --- | --- | --- | --- |
|  | OR | OR (95% CI) | OR (95% CI) | OR (95% CI) |  |
| NEU | 1 [Reference] | 1.07 (0.85-1.34) | 1.19 (0.95-1.50) | 1.62 (1.29-2.05) | <0.01 |
| LYM | 1 [Reference] | 0.95 (0.79-1.15) | 0.87 (0.72-1.04) | 0.99 (0.82-1.20) | 0.77 |
| MON | 1 [Reference] | 1.02 (0.82-1.28) | 1.20 (0.96-1.50) | 1.45 (1.15-1.84) | <0.01 |
| Hb | 1 [Reference] | 0.80 (0.63-1.01) | 0.83 (0.65-1.06) | 0.86 (0.64-1.15) | 0.30 |
| RDW | 1 [Reference] | 1.07 (0.84-1.37) | 1.44 (1.12-1.84) | 1.85 (1.51-2.28) | <0.01 |
| PLT | 1 [Reference] | 1.22 (1.01-1.47) | 1.28 (1.04-1.58) | 1.61 (1.31-1.97) | <0.01 |
| SAL | 1 [Reference] | 0.81 (0.66-0.99) | 0.76 (0.59-0.96) | 0.63 (0.48-0.83) | <0.01 |
| BMI | 1 [Reference] | 0.87 (0.70-1.07) | 0.76 (0.62-0.94) | 1.11 (0.88-1.40) | 0.25 |

Abbreviations: NEU, neutrophil; LYM, lymphocyte; MON, monocyte; Hb, hemoglobin; RDW, red cell distribution width; PLT, platelet; SAL, serum albumin; BMI, body mass index;

Model adjusted for age (20-39, 40-59, or ≥60 years), sex (male or female), race/ethnicity (non-Hispanic White, non-Hispanic Black or other race), marital status (married/living with partner, or single/divorced/widowed), education level (below high school, high school, or above high school), family PIR (≤1.0, 1.1–3.0, or >3.0), drinking status (nondrinker, former drinker, or current drinker), smoking status (never smoker, former smoker, or current smoker), physical activity (inactive, insufficiently active, or active), HEI (in quartiles), and CCI (continous).

**
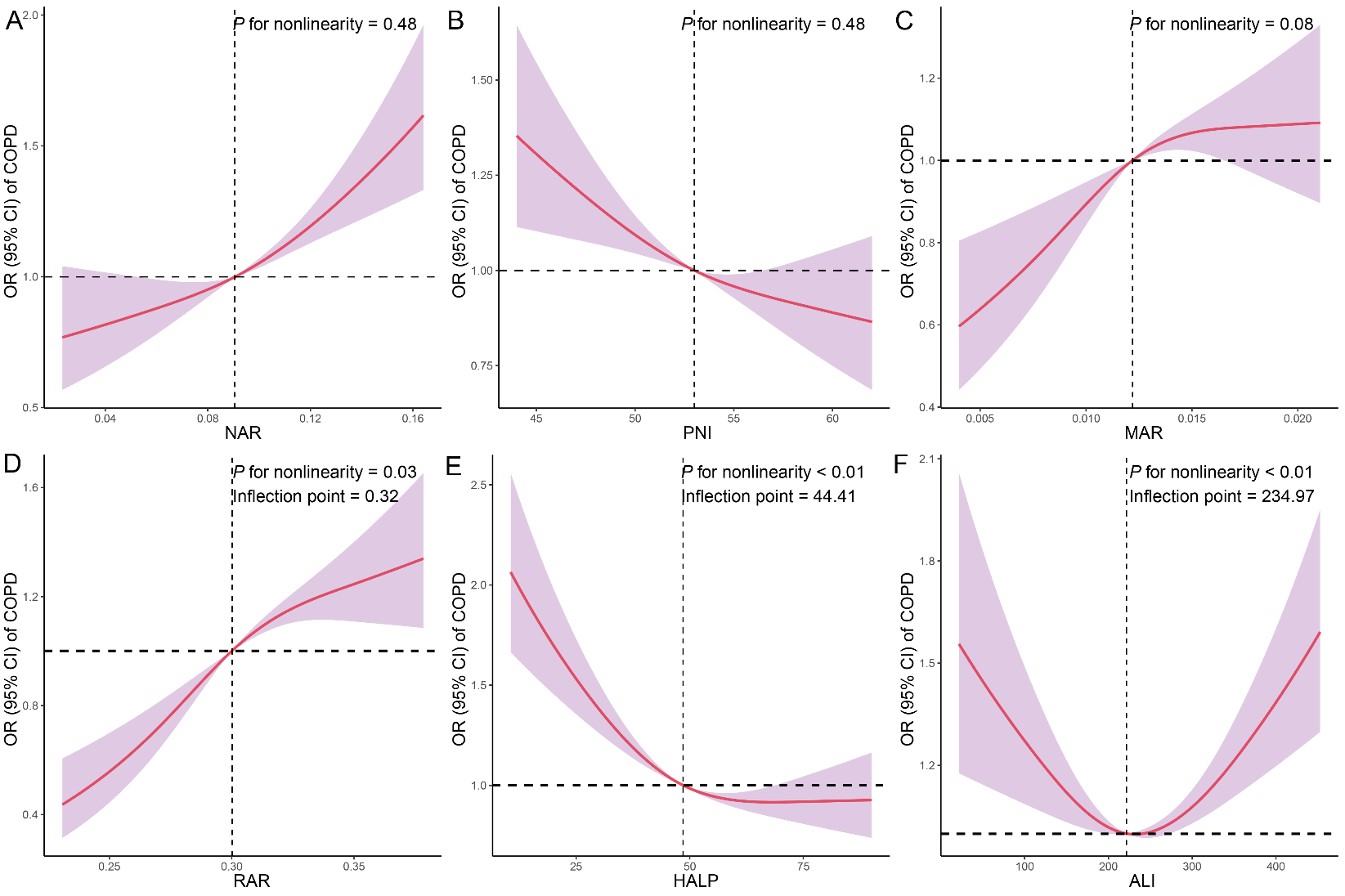
**

**Figure S2.** Restricted cubic spline (RCS) analysis with multivariate-adjusted associations of inflammation/nutrition‐based indicators (A: NAR; B: PNI; C: MAR; D: RAR; E: HALP; F: ALI; G: GNRI; and H: CONUT) with the prevalence of COPD among adults in NHANES 1999–2018. Models are adjusted for age (20-39, 40-59, or ≥60 years), sex (male or female), race/ethnicity (non-Hispanic White, non-Hispanic Black or other race), marital status (married/living with partner, or single/divorced/widowed), education level (below high school, high school, or above high school), family PIR (≤1.0, 1.1–3.0, or >3.0), drinking status (nondrinker, former drinker, or current drinker), smoking status (never smoker, former smoker, or current smoker), physical activity (inactive, insufficiently active, or active), HEI (in quartiles), and CCI (continous). NAR, neutrophil-albumin ratio; PNI, prognostic nutritional index; MAR, monocyte-albumin ratio; RAR, red cell distribution width-albumin ratio; HALP, hemoglobin, albumin, lymphocyte, and platelet; ALI, advanced lung cancer inflammation index; GNRI, geriatric nutrition risk index; CONUT, controlling nutritional status.

**Table S4.** COX regression analysis of the relationship between inflammation and nutritional indicators and all-cause mortality among adults with COPD in NHANES 1999–2018.

|  | Quartiles of inflammation and nutrition indicators | | | | *P_trend_* |
| --- | --- | --- | --- | --- | --- |
|  | OR | OR (95% CI) | OR (95% CI) | OR (95% CI) |  |
| NEU | 1 [Reference] | 0.93 (0.69-1.25) | 1.08 (0.77-1.53) | 1.28 (0.91-1.78) | 0.09 |
| LYM | 1 [Reference] | 0.74 (0.59-0.93) | 0.55 (0.42-0.73) | 0.65 (0.50-0.85) | <0.01 |
| MON | 1 [Reference] | 1.12 (0.84-1.49) | 0.93 (0.69-1.25) | 1.44 (1.06-1.94) | 0.04 |
| Hb | 1 [Reference] | 0.63 (0.48-0.83) | 0.62 (0.47-0.82) | 0.49 (0.37-0.63) | <0.01 |
| RDW | 1 [Reference] | 1.25 (0.97-1.60) | 1.39 (1.06-1.82) | 2.15 (1.59-2.92) | <0.01 |
| PLT | 1 [Reference] | 0.92 (0.74-1.15) | 0.78 (0.61-1.00) | 0.73 (0.57-0.95) | 0.02 |
| SAL | 1 [Reference] | 0.81 (0.61-1.07) | 0.65 (0.48-0.90) | 0.65 (0.49-0.87) | <0.01 |
| BMI | 1 [Reference] | 0.67 (0.50-0.89) | 0.66 (0.50-0.87) | 0.63 (0.46-0.87) | 0.01 |

Abbreviations: NEU, neutrophil; LYM, lymphocyte; MON, monocyte; Hb, hemoglobin; RDW, red cell distribution width; PLT, platelet; SAL, serum albumin; BMI, body mass index;

Model adjusted for age (20-39, 40-59, or ≥60 years), sex (male or female), race/ethnicity (non-Hispanic White, non-Hispanic Black or other race), marital status (married/living with partner, or single/divorced/widowed), education level (below high school, high school, or above high school), family PIR (≤1.0, 1.1–3.0, or >3.0), drinking status (nondrinker, former drinker, or current drinker), smoking status (never smoker, former smoker, or current smoker), physical activity (inactive, insufficiently active, or active), HEI (in quartiles), and CCI (continous).

**
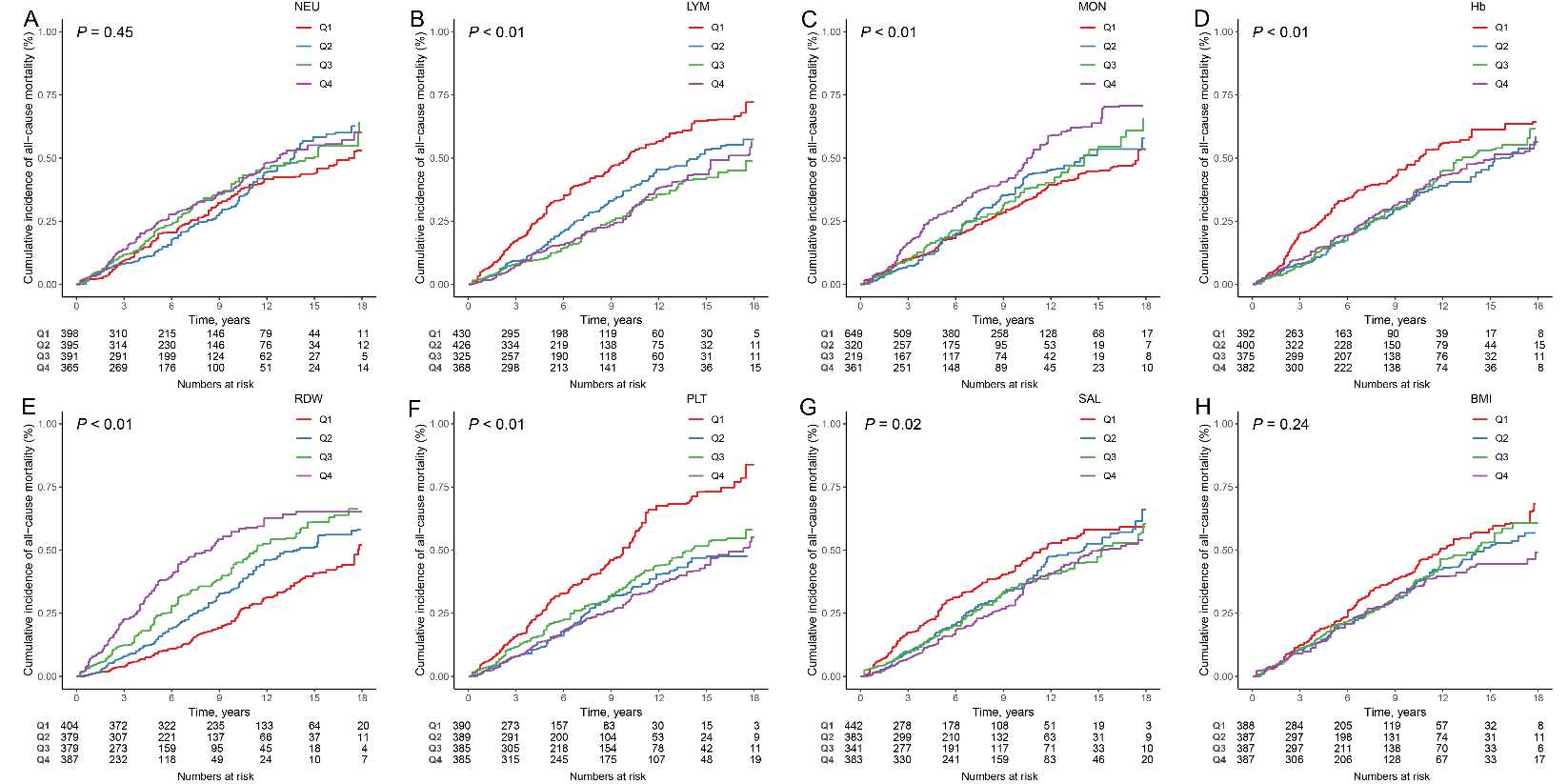
**

**Figure S3.** Kaplan-Meier survival curves for quartiles of inflammation and nutrition indicators (A: NEU; B: LYM; C: MON; D: Hb; E: RDW; F: PLT; G: SAL; and H: BMI) and mortality in adults. NEU, neutrophil; LYM, lymphocyte; MON, monocyte; Hb, hemoglobin; RDW, red cell distribution width; PLT, platelet; SAL, serum albumin; BMI, body mass index.


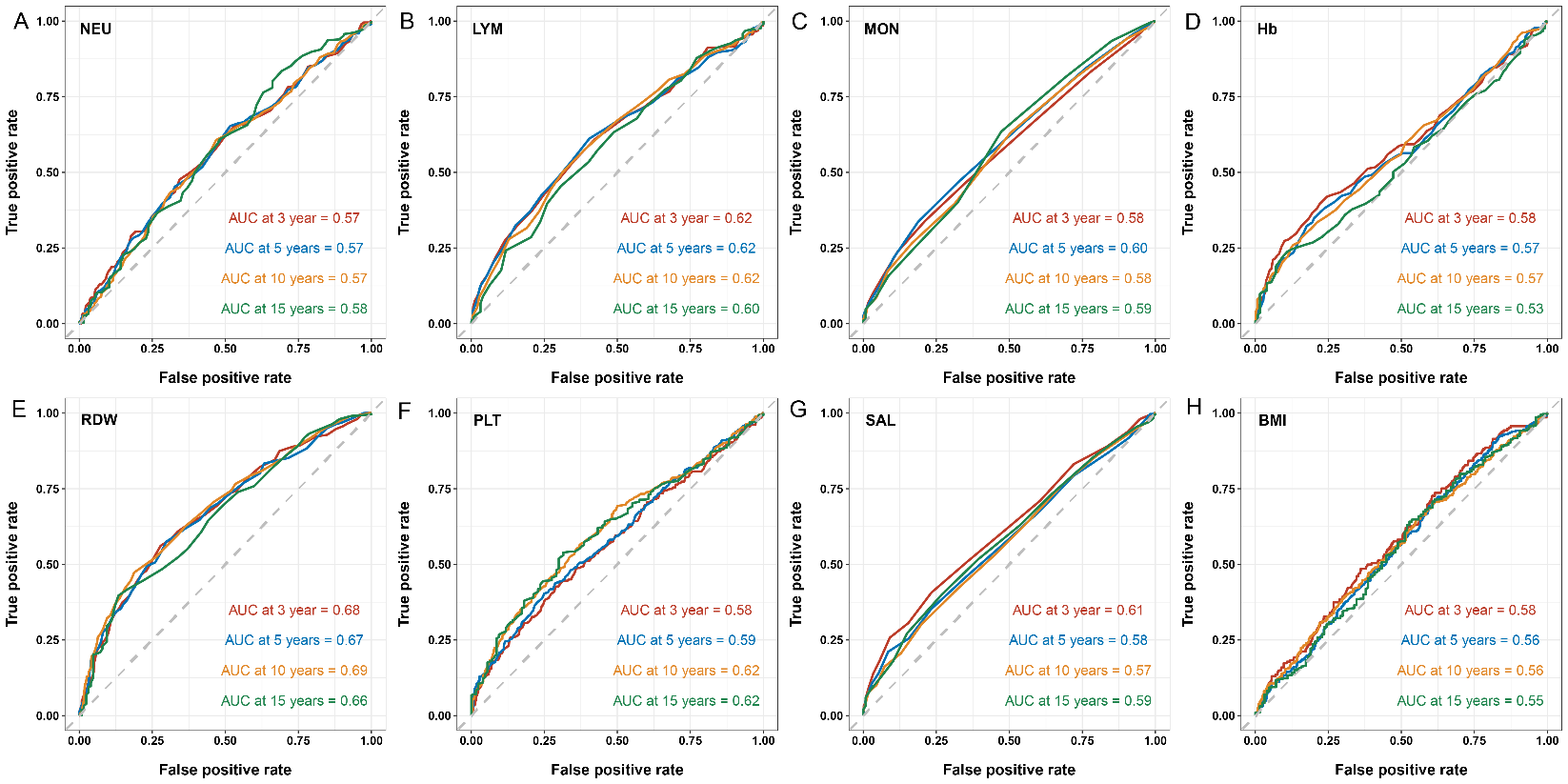


**Figure S4**. Predictive value of time-dependent ROC assessment of inflammation and nutritional indicators (A: NEU; B: LYM; C: MON; D: Hb; E: RDW; F: SAL; G: TC; and H: BMI) for 3-, 5-, and 10-year all-cause mortality. NEU, neutrophil; LYM, lymphocyte; MON, monocyte; Hb, hemoglobin; RDW, red cell distribution width; PLT, platelet; SAL, serum albumin; BMI, body mass index.
